# Tricked or trapped—Two decoy mechanisms in host–pathogen interactions

**DOI:** 10.1371/journal.ppat.1006761

**Published:** 2018-02-15

**Authors:** Judith K. Paulus, Renier A. L. van der Hoorn

**Affiliations:** The Plant Chemetics Laboratory, Department of Plant Sciences, University of Oxford, Oxford, United Kingdom; THE SAINSBURY LABORATORY, UNITED KINGDOM

Antagonistic interactions between hosts and pathogens frequently result in arms races. The host attempts to recognise the pathogen and inhibit its growth and spread, whereas the pathogen tries to subvert recognition and suppress host responses. These antagonistic interactions drive the evolution of ‘decoys’ in both hosts and pathogens. In host–pathogen interactions, the term decoy describes molecules that mimic a component at the host–pathogen interface that is manipulated during infection. Decoys undergo the same manipulation as the component they mimic, but they serve the opposite role, either by preventing manipulation of the component they mimic or by triggering a molecular recognition event. At least three different types of decoy have been defined, described in detail below. However, these different decoy models cause confusion on how they function mechanistically. Here, we discuss the three different types of decoys with examples and classify them according to two distinct mechanisms.

## Receptor decoys: Mimics to absorb ligands

**Fig 1:**
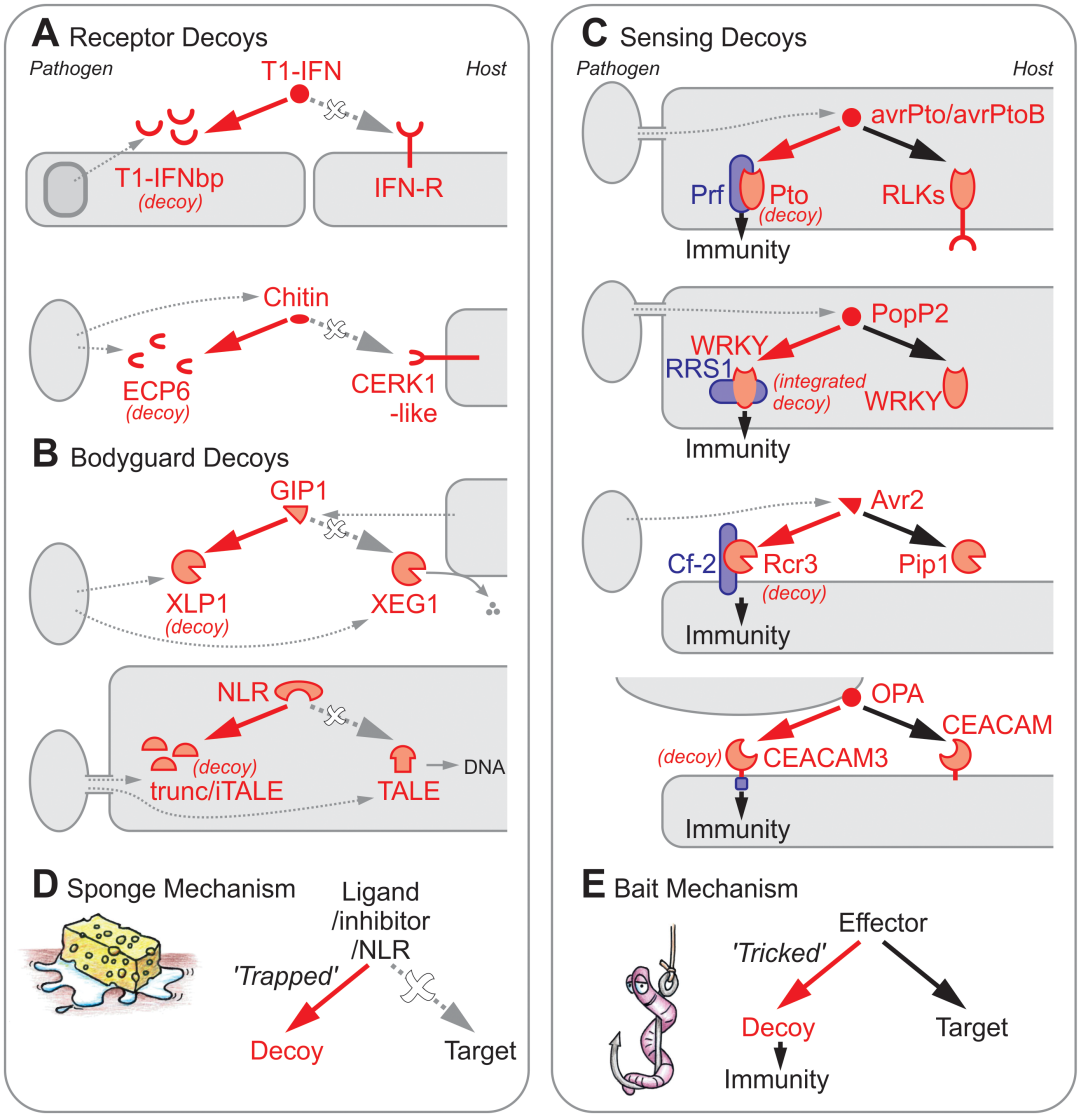
Three types of decoys act through two distinct mechanisms. Examples of Receptor (A), Bodyguard (B), and Sensing (C) decoys that act through either Sponge (D) or Bait (E) mechanisms. Avr2, Avirulence gene-2; avrPto, avirulence gene of *Pseudomonas syringae* pv. *tomato*; avrPtoB, avirulence gene B of *Pseudomonas syringae* pv. *tomato*; CEACAM, epithelial Carcino-embryonic Antigen-related Adhesion Molecules; CERK1, Chitin Elicitor Receptor Kinase-1; Cf-2, *Cladosporium fulvum* resistance gene-2; ECP6, extracellular Protein-6; GIP1, Glucanase Inhibitor Protein-1; NLR, Nod-like Receptor; OPA, opacity-associated membrane proteins; Pip1, Phytophthora-inhibited protease-1; PopP2, Pseudomonas outer protein P2; Prf, Pseudomonas resistance and fenthion sensitivity; Pto, Resistance to *Pseudomonas syringae* pv. *tomato*; Rcr3, Required for Cladosporium resistance-3; RLK, receptor-like kinase; RRS1, Resistance to Ralstonia solanacearum-1; T1-IFNbp, Type-1 interferon binding protein; TALE, Transcription Activator-like Effector; WRKY, Transcription factor with WRKY motif; XEG1, xyloglucan-specific endoglucanase-1; XLP1, XEG1-like protein-1.

Some pathogens use ‘Receptor decoys’ to interfere with host immune signalling (Fig 1A). Examples of Receptor decoys are found in large DNA viruses. Some viruses have acquired a diverse set of Receptor decoys through recombination events with the host [1]. These Receptor decoys typically encode for viral versions of receptor homologs of the host and bind chemokines or cytokines to prevent efficient immune signalling in the host. For example, ectromelia virus (causative of mouse pox) encodes the Type 1-interferon binding protein (T1-IFNbp), a Receptor decoy that is essential for its virulence [2]. T1-IFNbp mimics the interferon receptor and attaches to uninfected cells close to the infection site in liver and spleen. By binding T1-IFN, T1-IFNbp facilitates virus spread and impairs defence signalling [3]. Therefore, this virus-derived Receptor decoy absorbs T1-IFN, a key signal in host immune signalling.

A similar example of a pathogen-derived Receptor decoy is extracellular Protein-6 (Ecp6), a Lysin Motif (LysM)-containing effector that is secreted by the fungal pathogen *Cladosporium fulvum* during infection of tomato plants. Ecp6 suppresses chitin recognition and is therefore instrumental for *C*. *fulvum* virulence [4]. Chitin is an essential component of fungal cell walls, and many plants can sense fungal chitin through LysM-containing receptors such as Chitin Elicitor Receptor Kinase-1 (CERK1) and its homologs. Interestingly, Ecp6 captures chitin oligomers with high affinity and is thought to outcompete the LysM-based host immune receptor for chitin binding [5]. Therefore, Ecp6 mimics the chitin-binding capacity of the receptor and acts as a Receptor decoy by binding chitin to prevent recognition by the host. Interestingly, LysM-based effectors are widespread amongst fungal plant pathogens, so chitin absorption by LysM effectors appears to be a commonly used decoy strategy [6].

## Bodyguard decoys: Protecting secreted virulence factors

Some pathogens employ ‘Bodyguard decoys’ to protect virulence factors [7]. Bodyguard decoys are inactive mimics of secreted virulence factors. They accompany these virulence factors and efficiently bind host-derived defence proteins that aim to suppress these virulence factors (Fig 1B). For instance, soybean secretes inhibitor *Gm*GIP1 that strongly inhibits the xyloglucan-specific endoglucanase *Ps*XEG1 of the soybean oomycete pathogen *Phytophthora sojae* [8]. *Ps*XEG1 is an important virulence factor that probably acts on the host cell wall during infection. *P*. *sojae*, however, protects *Ps*XEG1 by cosecreting the Bodyguard decoy *Ps*XLP1, a truncated paralog of *Ps*XEG1 with no known enzymatic activity [8]. *Ps*XLP1 has a higher binding affinity for *Gm*GIP1 and acts as a Bodyguard decoy by outcompeting the inhibition of *Ps*XEG1.

A similar Bodyguard decoy concept has been proposed for truncated versions of Transcription Activator-like Effectors (TALEs) that are secreted by the bacterial plant pathogen *Xanthomonas* [7]. TALEs *trans*-activate host genes in the plant cell nucleus to facilitate bacterial infection and therefore have a major role in virulence. Some host plants carry Nod-like Receptor (NLR) proteins that confer recognition of TALEs and trigger immune responses. Remarkably, a recently discovered class of truncated TALEs named ‘iTALEs’ [9] or ‘truncTALEs’ [10] with N- and C-terminal deletions can suppress TALE recognition by these NLRs, possibly by binding the NLR without activating it. Therefore, these truncated TALEs may act as a Bodyguard decoy to prevent NLR activation through full-length TALEs that act as virulence factors.

## Sensing decoys: Mimics of effector targets acting as coreceptors

The decoy concept has also been frequently used to explain the indirect recognition mechanisms through products of disease resistance genes in plants [11]. The usual interpretation is that these resistance genes monitor the modification of a decoy that mimics the target of a pathogen-derived effector. These ‘Sensing decoys’ act as coreceptors with resistance gene products (Fig 1C).

A classic example of a Sensing decoy is the tomato resistance gene product Pto. Pto is a serine/threonine (Ser/Thr) kinase that confers resistance to strains of the bacterial pathogen *Pseudomonas syringae* secreting the Type-III effectors AvrPto and AvrPtoB [12,13]. AvrPto and AvrPtoB target receptor-like kinases (RLKs) involved in immune signalling by inhibiting or ubiquitinating them, respectively. Pto mimics these RLKs and confers recognition of AvrPto and AvrPtoB together with its binding partner Pseudomonas resistance and fenthion sensitivity (Prf), an NLR that triggers immune signalling. PBS1 is a similar Sensing decoy in the model plant *Arabidopsis thaliana* [14]. As with Pto, PBS1 is a Ser/Thr kinase that detects AvrPphB, a Type-III effector of *P*. *syringae*. AvrPphB is a cysteine protease that cleaves the kinase domain of immune-related RLKs. PBS1 is a Sensing decoy that mimics the target of AvrPphB and confers recognition of this effector by activating its binding partner Resistance to *Pseudomonas syringae*-5 (RPS5), an NLR that triggers immune signalling [14].

It was recently discovered that many plant NLRs may carry a Sensing decoy within themselves. For instance, the NLR Resistance to Ralstonia solanacearum-1 (RRS1) from *A*. *thaliana* carries like a WRKY-DNA–binding domain [15], and the NLRs RGA5 and Pik-1 in rice contain a heavy metal–associated (HMA) domain related to ATX1 (RATX1) [16,17]. These domains seem to mimic targets of effectors and enable pathogen detection. Therefore, they were named ‘Integrated decoys’ [18]. However, given that the specific biochemical activities of the ancestral effector targets and their NLR-integrated counterparts are generally unknown, they could be sensor domains retaining their biochemical activity as an extraneous domain within a classic NLR architecture [19].

Not all Sensing decoys associate with NLRs. A classic example comes from a study of the *Cladosporium fulvum* resistance gene-2 (*Cf-2*) resistance gene of tomato, which encodes a transmembrane receptor-like protein. Cf-2 confers recognition of the avirulence 2 (Avr2) effector secreted by the fungal tomato pathogen *C*. *fulvum*. Avr2 contributes to virulence by inhibiting Phytophthora-inhibited protease-1 (Pip1) and other extracellular papain-like Cys proteases of tomato. Cf-2 perceives Avr2 through its coreceptor Required for Cladosporium resistance-3 (Rcr3), a paralog of Pip1, which acts as a Sensing decoy to confer Avr2 recognition [20].

Likewise, human epithelial carcinoembryonic antigen-related adhesion molecules 3 (CEACAM3) can be considered to be a Sensing decoy that acts during gonorrhoea infection. To facilitate close attachment to epithelial cells in the human urogenital tract, the bacterial pathogen *Neisseria gonorrhoeae* expresses opacity-associated (Opa) membrane proteins [21]. Opas interact with a different human CEACAM, and this Opa–CEACAM interaction triggers bacterial engulfment and transcytosis and thereby facilitates infection [22]. However, some Opas also bind to the decoy CEACAM3, and this Opa–CEACAM3 interaction triggers efficient phagocytosis of the bacteria and recruitment and downstream activation of the neutrophils’ antimicrobial responses, including degranulation and oxidative burst [23]. Therefore, CEACAM3 acts as a Sensing decoy that allows the capture and killing of CEACAM-targeting microbes.

The concept of Sensing decoy can be extended beyond proteins. TALEs such as AvrBs3 from *X*. *campestris* and AvrHah1 from *X*. *gardneri* reprogram the host by binding and activating promoters of *upa* (up-regulated by AvrBs3) and other genes in the host [24,25]. The promotor of the pepper resistance gene *Bs3* (*pBs3*) mimics the targets of these TALEs and transcriptionally activates the *Bs3* gene product, leading to a localised cell death response that stops further pathogen growth. Therefore, *pBs3* acts as a nonprotein Sensing decoy to trick AvrBs3 and AvrHah1 into a recognition event [25,26].

## Two decoy mechanisms: Sponge and bait

The above examples of Receptor, Bodyguard, and Sensing decoys illustrate that the decoy concept is discussed frequently in host–pathogen interactions. This, however, causes confusion in the field because not all these decoys are mechanistically the same.

Receptor decoys are expected to have a higher affinity and/or abundance when compared to the receptor they mimic, to prevent the ligands from reaching the receptors and inducing immune signalling. Likewise, Bodyguard decoys must have a higher affinity and/or abundance when compared to the acting virulence factor to prevent the virulence factor from being inactivated or recognised. Therefore, both Receptor and Bodyguard decoys act as a sponge to absorb (Fig 1D). The ligand or virulence factor, respectively, is ‘trapped’ because it cannot reach its operative target as it is captured by the Sponge mechanism.

In contrast, all Sensing decoys act like a bait. These baits are not necessarily preventing the interaction of the effector with its operative target. The response to recognition can simply overrule the benefits of the effector manipulating its operative target. Therefore, in the Bait mechanism, the effector is ‘tricked’ by the Sensing decoy that prompts a recognition event (Fig 1E). Indeed, there is no evidence that Sensing decoys like Pto, PBS1, HMA, Rcr3, CEACAM3, and pBs3 prevent the interaction of the sensed effector with its operative target.

## Further thoughts

Sponge and Bait mechanisms occur frequently at the host–pathogen interface. By its definition, decoys are thought to have no additional role, e.g., in development, disease or resistance. Hypothetically, however, because of their crucial role, decoys can become an attractive target for manipulation and can evolve into a target. In addition, also outside of that specific host–pathogen interaction, decoys may play a role. Therefore, it is important to use decoy terminology when the decoy acts in conjunction with the component they mimic.

Interestingly, the presented examples indicate a trend: all Sponge mechanisms that we define here are pathogen derived, while Bait mechanisms are host derived. There is, however, no reason to exclude the existence of host-derived Sponge mechanisms. For instance, the absorbance of pathogen-derived toxins to prevent them from reaching their target in the host is likely to occur. Bait mechanisms may only be host-derived because invading pathogens are more likely to sense the host in a direct way, not least because receptors that recognize the host are also under selection pressure and coevolve with the host. Because some pathogenic organisms may become a host themselves, it is conceivable that they may also have decoys that act as a bait.

While both types of decoy mechanisms have been described in the literature, much remains to be discovered. The discovery of more decoy examples will help us to find novel drug targets as well as new possibilities to improve host immunity. The latter is illustrated by a broader resistance spectrum upon decoy engineering of PBS1 in *Arabidopsis* plants [27].
